# Cytomegalovirus Infection in Infancy May Increase the Risk of Subsequent Epilepsy and Autism Spectrum Disorder in Childhood

**DOI:** 10.3390/children8111040

**Published:** 2021-11-11

**Authors:** Chien-Heng Lin, I.-Ching Chou, Inn-Chi Lee, Syuan-Yu Hong

**Affiliations:** 1 Division of Pediatrics Pulmonology, China Medical University Children’s Hospital, Taichung 404327, Taiwan; lch227@ms39.hinet.net; 2 Department of Biomedical Imaging and Radiological Science, College of Medicine, China Medical University, Taichung 404327, Taiwan; 3 Division of Pediatrics Neurology, China Medical University Children’s Hospital, Taichung 404327, Taiwan; iching@mail.cmu.edu.tw; 4 Department of Pediatrics, Chung Shan Medical University Hospital, and Institute of Medicine, School of Medicine, Chung Shan Medical University, Taichung 402306, Taiwan; y610@mercury.csmu.edu.tw; 5 Department of Medicine, School of Medicine, China Medical University, Taichung 404328, Taiwan; 6 Institute of Biomedicine, School of Medicine, China Medical University, Taichung 404328, Taiwan

**Keywords:** CMV infections, epilepsy, infancy, attention deficit hyperactivity disorder, autism spectrum disorder

## Abstract

Cytomegalovirus (CMV) is a ubiquitous virus, and CMV-associated diseases range from mild illness in immunologically normal hosts to life-threatening diseases in newborns and immunocompromised children. This study investigated the association between childhood CMV infection and subsequent epilepsy or neurodevelopmental disorders, attention deficit hyperactivity disorder (ADHD), and autism spectrum disorder (ASD). A retrospective analysis was performed on data for 69 children with confirmed CMV infections (CMV infection group) and 292 patients with other infections (control group) between 1 January 2006 to 31 December 2012. The results indicated that the CMV infection group had a higher risk of epilepsy in comparison to the control (odds ratio (OR), 16.4; 95% CI (confidence interval), 3.32–80.7; *p* = 0.001). Epilepsy risk increased in younger children (age 0–2) with CMV infection when compared to the control group (OR, 32.6; 95% CI, 3.84–276; *p* = 0.001). The ASD risk was also determined to be higher in the CMV infection group (OR, 17.9; 95% CI, 1.96–162; *p* = 0.01). The ADHD risk between the groups was not significant. This study suggests that CMV infection in infancy may increase the risk of subsequent epilepsy and ASD, especially in infants younger than 2 years, but is not associated with ADHD.

## 1. Introduction

Cytomegalovirus (CMV) infection is an inconspicuous and mostly symptomless disease commonly infecting people of all ages throughout the world. In developing countries, most children are infected by 3 years of age; by contrast, in developed countries, infection can often occur throughout childhood and adolescence, where as much as 60% to 80% of some countries’ populations are infected with CMV by adulthood [1].

Only approximately 10% of acquired CMV infections produce symptoms. These include mononucleosis-like syndrome, fever, fatigue, pharyngitis, adenopathy (especially cervical adenopathy), hepatitis, and hepatosplenomegaly (HSM) [2]. In the pediatric population, CMV infection–related diseases range from asymptomatic or mild diseases to severe and life-threatening diseases, which affect newborns, immunologically normal hosts, and immunocompromised children [3].

Congenital CMV infection often results in nonhereditary sensorineural hearing loss or other long-term neurodevelopmental disorders (NDDs). Young children who survive a CMV infection congenitally might have a higher risk of developing neurodevelopmental sequelae, including intellectual disability, cerebral palsy, hearing loss, and seizures [4].

Different ages of initial CMV infection and different immune statuses in infants and children might imply divergent clinical manifestations and long-term outcomes. The relationship between congenital infection and NDD is currently poorly understood [5,6,7]. Therefore, our hypothesis is that CMV infection during infancy may increase the risk of subsequent epilepsy and certain NDDs.

This study aimed to retrospectively investigate the relationship between CMV infection in children of different ages and the incidence of epilepsy and other associated NDDs, such as attention deficit hyperactivity disorder (ADHD) and autism spectrum disorder (ASD).

## 2. Materials and Methods

The protocol used in this study was approved by the China Medical University Ethics Committee (CMUH107-REC2-152). The data of children aged <18 years who had definite CMV infections between 1 January 2006 and 31 December 2012 were collected retrospectively. Various laboratory tests were used to confirm CMV [1]. Confirming congenital CMV infection required at least two positive CMV polymerase chain reaction (PCR) results using urine obtained within the first 3 weeks of life [2]. The confirmation of early postnatal infection required at least two positive urine CMV PCR results taken after the first 3 weeks of life in addition to a negative result obtained from the newborns’ dried blood spot samples for the purposes of screening [3]. For immunocompetent children, an acute or recent CMV infection was verified with a CMV Immunoglobulin M (IgM) antibody or with a positive CMV quantitative polymerase chain reaction (PCR) assay of the blood. We then comprehensively reviewed the respective medical records up to the end of 2017 to investigate whether a first diagnosis of epilepsy, ASD, or ADHD after CMV infection was confirmed. The final analysis excluded individuals who had had more than one CMV infection episode, who had died during follow-up, who had been diagnosed with neoplasms, who had undergone organ transplantation, or who had suffered from any serious disease affecting the immune system (e.g., systemic lupus erythematosus or aplastic anemia) before or after CMV infection. The immunocompetent children enrolled in the study were relatively healthy; that is, they had no epilepsy or neurologic, metabolic, autoimmune, or congenital disorders before the onset of CMV infection.

A total of 69 patients met the criteria for this study. The patients were diagnosed with epilepsy by a pediatric neurologist if they had displayed two unprovoked seizures separated by at least 24 h. In addition, the patients were diagnosed with ASD and ADHD if they met the required diagnostic criteria as presented in the fourth and fifth editions of the Diagnostic and Statistical Manual of Mental Disorders (DSM-4, DSM-5) [8,9]. These diagnoses were made by either a pediatric psychiatrist or a neurologist at the China Medical University Children’s Hospital in an inpatient or outpatient capacity. For the matched control group (age and mean follow-up years), a total of 292 children was identified between 1 January 2006, and 31 December 2012. These children had non-CMV infections (e.g., acute tonsillitis, acute gastroenteritis, urinary tract infection) that were diagnosed through positive culture results from stool, urine, throat, or other discharges but that were negative from blood or cerebrospinal fluid (CSF). Confounding factors in this study were labor condition, sex, and age of infection onset.

The CMV infection group and control group were divided into four subgroups determined by the age of infection onset: infants and young toddlers (ages 0–2 years), preschoolers (ages 2–5 years), young children (ages 5–10 years), and young teens and teenagers (ages >10 years) (Table 1).

## 3. Statistical Analysis

The categorical variables between groups were analyzed using χ2 tests. The incidence density rates of ASD, ADHD, and epilepsy were calculated for the CMV infection group (subdivided into age intervals and CMV-associated symptoms) and the control group. The hazard ratios and 95% confidence intervals (CIs) of ASD, ADHD, and epilepsy for the CMV infection group in relation to the control group were estimated using Cox proportional hazards regression.

PASW Statistics v18.0 software (SPSS Inc., Chicago, IL, USA) was used for the statistical analyses. The results were deemed statistically significant if the two-tailed *p*-values were <0.05.

## 4. Results

### 4.1. Data Analysis

A total of 69 children with CMV infections (CMV infection group) and 292 children with non-CMV infections (control group) diagnosed between 1 January 2006 and 31 December 2012 were enrolled.

The mean age of the participants was 3.78 years (standard deviation 4.66), and there was a higher proportion of boys than girls (55.1% vs. 44.9%). The demographic data are summarized in Table 1.

**Table 1:** Demographic and clinical data for the cytomegalovirus (CMV) infection and control groups.

Demographic Data	CMV Infection, *n* = 69 (%)	Controls, *n* = 292 (%)	*p*-Value
Mean age of infection (years) (SD)	3.78 (4.66)	3.62 (4.50)	0.90
Gender			0.27
Male	38 (55.1)	182 (62.3)	
Female	31 (44.9)	110 (37.7)	
Labor			0.68
Preterm	7 (10.1)	25 (8.6)	
Term	62 (89.9)	267 (91.4)	
Stratified by age (years)			0.18
0–2	33 (47.8)	122 (41.8)	
2–5	19 (27.5)	89 (30.5)	
6–10	9 (13.0)	63 (21.6)	
>10	8(11.6)	18 (6.2)	
Associated diseases or symptoms			
SGA	5 (7.2)	-	
Thrombocytopenia	5 (7.2)	-	
Mononucleosis-like syndrome	11 (15.9)	-	
Prolonged fever	9 (13.0)	-	
Neurological manifestations	9 (13.0)	-	
Hepatitis and/or HSM	28 (40.6)	-	
Myocarditis and sepsis-like syndrome	6 (8.7)	-	
Skin manifestations	2 (2.9)	-	

SGA, small gestational age; HSM, hepatosplenomegaly; SD, standard deviation.

**Figure 1:**
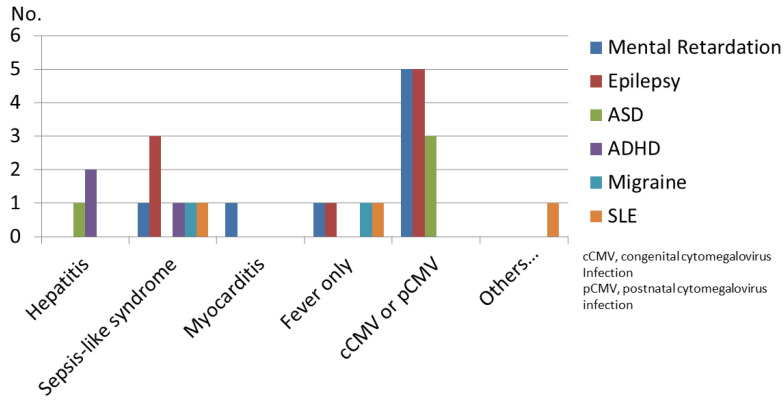
Clinical manifestations in children with cytomegalovirus (CMV) infection before 2 years of age. SGA: small for gestational age. ASD: autism spectrum disorder, ADHD: attention deficit hyperactivity disorder, SLE: systemic lupus erythematosus, Neurological manifestations indicate microcephaly, intracranial calcifications, sensorineural hearing loss, seizures, and other brain structural abnormalities; skin manifestations indicate petechial rash, jaundice, and arthralgias.

The CMV-associated symptoms in the study were classified as small for gestational age (SGA) (*n* = 5, 7.2%), thrombocytopenia (*n* = 5, 7.2%), mononucleosis-like syndrome (*n* = 11, 15.9%), prolonged fever (*n* = 9, 13%), neurological manifestations (*n* = 9, 13%), hepatitis or HSM (*n* = 8, 40.6%), myocarditis and sepsis-like syndrome (*n* = 6, 8.7%), and skin manifestations (*n* = 2, 2.9%). In addition, the CMV-associated clinical manifestations for children younger than 2 years of age are summarized in Figure 1.

### 4.2. Neurodevelopmental Outcomes

Table 2 compares the group for CMV infection at age 0–2 years and the control group in terms of their incidence rates and relative risks of epilepsy, ASD, and ADHD. The overall epilepsy risk in the CMV infection group was higher than in the control group (OR, 16.4; 95% CI, 3.32–80.7; *p* = 0.001). Furthermore, the risk of epilepsy in the group for CMV infection at age 0–2 years was even higher than in the control (OR, 32.6; 95% CI, 3.84–276; *p* = 0.001). The overall risk of ASD in the CMV infection group was higher than in the control group (OR, 17.9; 95% CI, 1.96–162; *p* = 0.01). In addition, the risk of ASD in the group for CMV infection at age 0–2 years was higher than that in the control (OR, 12.1; 95% CI, 1.21–120; *p* = 0.03). However, the CMV infection group and the control group exhibited no significant difference in incidence rates or relative risks for ADHD (OR, 1.01; 95% CI, 0.22–5.1; *p* = 0.94).

### 4.3. Neurodevelopmental Disabilities in Children with CMV Infection

We summarized the clinical manifestations of patients with CMV infection who developed epilepsy or ASD (Table 3). Of the nine patients, eight children were aged younger than 2 years, and the most common etiology was congenital CMV infection (55.5%, *n* = 5), followed by immunocompetence (*n* = 3, 33.3%); two children with congenital CMV infection had both epilepsy and ASD in childhood. Children with congenital CMV infections had poorer neurological outcomes than those whose CMV infection occurred through other means.

## 5. Discussion

In this retrospective study, children with CMV infection had a significantly increased incidence rate of subsequent epilepsy and ASD compared with those without CMV infection, especially infants younger than 2 years; however, the subsequent ADHD incidence rate of those with CMV infection was not significantly increased.

Long-term neurological problems in children with congenital CMV disease present a serious problem because of the associated risk of hearing loss, eye diseases, intellectual disability, cerebral palsy, and seizures [10,11]. Some evidence also suggests that central nervous system (CNS) infections in fetuses or in those in the earliest stages of life compromise early development of the CNS and might raise the risk of epilepsy and other NDDs later in life, which would then require special educational support and therapy (physical, occupational, language, or speech); this imposes heavy burdens on not only individuals but also families and society in general [12,13]. Therefore, this study explored neurodevelopmental outcomes in children and infants with confirmed CMV infections and proposed an association between CMV infection in infancy and the subsequent development of epilepsy and ASD in childhood. However, the study observed no significant difference between the risk of ADHD for the control population and for children and infants with confirmed CMV infection.

Our study results also found that CMV-related presentations varied during different periods between birth and 2 years of age. In congenital CMV infections, neurological symptoms and SGA were dominant, followed by myocarditis and sepsis-like syndrome. Non-neurological symptoms were prevalent in the group of immunocompetent children and those with early postnatal infection (Figure 1). We proposed two hypotheses: first, that HSM or hepatitis were less prominent than neurological symptoms, myocarditis, or sepsis-like syndrome in the first few days of life. Second, we proposed that CMV, as a neurotropic virus, can activate inflammatory pathways causing the release of various proinflammatory biomarkers and may lead to immediate or delayed neuropathological alterations within human brains in the early stages of development. In other words, neonatal CNS is more vulnerable to CMV infection, which can irreversibly disrupt the complex structural and functional architecture of the CNS, causing pronounced neurological symptoms early on and leading to long-term neurodevelopmental impairment in the development of epilepsy and ASD [14,15].

The relationship between congenital CMV infection and the risk of epilepsy has been investigated previously [14,15,16]. In addition, brain MRI abnormalities in these patients have been linked to later epilepsy and poor developmental outcomes [17,18]. This study arrived at the same results. We inferred that poor developmental outcomes and epilepsy resulted from potential damage to early brain development caused by CMV. However, human brains experience a rapid growth phase between birth and age 2 years [19]; therefore, we presumed that CMV infection beyond the fetus stage and throughout infancy could also impede brain formation and thus increase the risk of NDDs. For this reason, we chose 2 years as the cutoff age in this study. Moreover, all the patients with epilepsy in our study group were younger than 2 years; three of them (3/7, 42%) had non-congenital infections. Furthermore, three out of four patients developed ASD during infancy, and three of them had congenital CMV infections. The remaining patient, an immunocompetent patient infected with CMV at age 2.1, was diagnosed with ASD at age 3 (Table 3). Studies disagree over whether early postnatal CMV infection adversely affects long-term neurodevelopmental outcomes in preterm infants. Based on the data from follow-up studies comparing CMV-infected infants with noninfected infants, no differences were observed in terms of growth, hearing, or cerebral palsy [6,20,21,22]. One study revealed that postnatal CMV infection in preterm children had no impact on motor development, and no child developed sensorineural hearing loss within the first 6 years of life [6]. Although our study demonstrated a similar trend, namely that patients with non-congenital CMV in infancy, regardless of infection type, exhibited less severe neurological sequelae, we suggested an association between early CMV infection and the development of epilepsy and ASD by analyzing our patients and by referring to research publications [8,23,24,25,26]. However, more research is required to support our observations. A prospective cohort longitudinal study should be designed for both the CMV infection group and control group in order to investigate whether the incidence of neurodevelopment disorders is significantly different between these two groups during long-term follow-up, to support our observation.

The exact pathophysiology of childhood CMV infection that raises the risk of subsequent ASD is not clear; this is mostly due to the lower case numbers in autopsy studies and limited related animal models about brain development [27]. Microglia are macrophages that reside in the central nervous system and perform specialized roles in controlling infection, removing cellular debris, and supporting tissue homeostasis [28]. Thus, disruptions in the microbiome may affect microglial developmental programs, driving neurodevelopmental l phenotypes, such ASD or schizophrenia [26].

Our study was subject to several limitations: first, despite a high prevalence of congenital CMV infection in ASD cases, few events (four events) in the study imposed a serious limitation. Second, several noninfectious factors that were not investigated in this study might have been confused with epilepsy and ASD development in childhood. These factors include structural or metabolic disorders, neurogenetic factors, and immune status. Finally, this retrospective study drew upon the data collected from a single medical center, excluding patients who may have attended a different hospital or clinic. This factor may have impacted the results.

## 6. Conclusions

This study assessed the possible effects of CMV infection in infancy. We found that the earlier infant CMV infections occur, the higher the risk of the infant developing epilepsy and ASD in the future, especially those who are younger than 2 years. Therefore, identifying and assessing epilepsy and other NDDs in children at an early stage facilitates subsequent intervention and treatment programs. Future studies should include an examination of whether a relationship exists between CMV infection and other NDDs, such as Tourette syndrome, speech and language disorders, and communication disorders. Future research on the mechanism through which CMV infection increases the risk of epilepsy and ASD is warranted.

## Figures and Tables

**Table 2:** Incidence rates and relative risks (odds ratio) of epilepsy, autism spectrum disorder (ASD), and attention deficit hyperactivity disorder (ADHD) for controls, for populations of individuals with cytomegalovirus (CMV) Infections, and individuals with CMV infections at age 0–2 years using a logistic regression model.

Group	Epilepsy				ASD				ADHD			
	Event (No.)	IR(%)	OR (95% CI)	*p*	Event (No.)	IR(%)	OR (95% CI)	*p*	Event (No.)	IR(%)	OR (95% CI)	*p*
Controls (*n* = 292)	2	0.68	reference		1	0.34	reference		8	2.73	reference	
CMV group (*n* = 69)	7	10.1	16.4 (3.32–80.7)	0.001	4	5.8	17.9 (1.96–162.8)	0.01	2	2.89	1.06 (0.22–5.10)	0.94
CMV infection at age 0 to 2 years (*n* = 33)	7	21.2	32.6 (3.84–276.2)	0.001	3	9.1	12.1 (1.21–120.5)	0.03	0	0	N/A	>0.99

IR, Incidence rate; OR, Odds ratio; CI, Confidence interval.

**Table 3:** Clinical Summaries of Patients with CMV Infections Who Developed Epilepsy or ASD.

Patient No.	Sex/Labor	Age of CMV INF	Type of Infection	Antiviral Treatment	MR	Epilepsy/ Age of Dx	ASD/ Age of Dx	Last EEG Patterns/Age	Number of AED Uses	Seizure Control/Freq	Brain MRI
CMV01	F/P	0	Congenital	GCV	S	+/1 y	-	H yps/2 y	4	Unfavorable	WM
CMV02	M/T	0	Congenital	GCV	M to S	+/1 y	+/3 y	Multi/5 y	1	Controlled	VM
CMV03	M/T	0	Congenital	GCV	M	+/1 y	+/2 y	Multi/2 y	1	Controlled	HCP
CMV04	F/T	0	Congenital	GCV	S	+/1 y	-	Multi/3 y	1	Controlled	HCP
CMV05	M/T	0	E/P	-	-	+/2 y	-	Normal/9 y	1	Controlled	Normal
CMV06	M/T	1.5 y	Immunocompetent	-	-	+/2 y	-	Normal/8 y	0	Yearly	Normal
CMV07	F/T	1 y	Immunocompetent	-	M to S	+/1 y	-	Multi/2 y	2	Monthly	WM
CMV08	F/P	0	Congenital	GCV	Mild	-	+/3 y	N/A	N/A	N/A	Normal
CMV09	F/T	2.1 y	Immunocompetent	-	-	-	+/3 y	N/A	N/A	N/A	Normal

M, male; F, female; AED, Antiepileptic drug; E/P, Early postnatal; Dx, diagnosis; EEG, Electroencephalography; Freq, frequency; GCV, ganciclovir; +, present; -, not present; HCP, hydrocephalus; Hyps, hypsarrhythmia; INF, infection; MR, mental retardation; MRI, magnetic resonance imaging; M, moderate; Multi, multifocal spikes; N/A, not applicable; P, preterm; S, severe; T, term; VM, ventriculomegaly; WM, white matter lesions; y, years.

## Data Availability

Datasets are available on request.

## Abbreviations

ADHD	Attention Deficit Hyperactivity Disorder
ASD	Autism Spectrum Disorder
CI	Confidence Interval
DSM	Diagnostic and Statistical Manual of Mental Disorders
FS	Febrile Seizure
MR	Mental Retardation
NDDs	Neurodevelopmental Disorders
OR	Odds Ratio
TS	Tourette Syndrome
